# Does cardiac imaging surveillance strategy influence outcomes in patients with early breast cancer?

**DOI:** 10.3389/fonc.2023.1168651

**Published:** 2023-06-27

**Authors:** Kai Yi Wu, Sarah Parent, Lingyu Xu, Maryam Yaqoob, W. Allan Black, Andrea Shysh, John R. Mackey, Karen King, Harald Becher, Edith Pituskin, D. Ian Paterson

**Affiliations:** ^1^ Mazankowski Alberta Heart Institute, University of Alberta, Edmonton, AB, Canada; ^2^ Royal University Hospital, University of Saskatchewan, Saskatoon, SK, Canada; ^3^ Cardiovascular Medicine Division, University of Pennsylvania School of Medicine, Philadelphia, PA, United States; ^4^ Faculty of Nursing, University of Alberta, Edmonton, AB, Canada; ^5^ Cross Cancer Institute, University of Alberta, Edmonton, AB, Canada; ^6^ University of Ottawa Heart Institute, University of Ottawa, Ottawa, ON, Canada

**Keywords:** cardiac imaging, breast cancer, cardiotoxicity, surveillance, survival

## Abstract

**Background:**

Many patients with breast cancer receive therapies with the potential to cause cardiotoxicity. Echocardiography and multiple-gated acquisition (MUGA) scans are the most used modalities to assess cardiac function during treatment in high-risk patients; however, the optimal imaging strategy and the impact on outcome are unknown.

**Methods:**

Consecutive patients with stage 0-3 breast cancer undergoing pre-treatment echocardiography or MUGA were identified from a tertiary care cancer center from 2010-2019. Demographics, medical history, imaging data and clinical events were collected from hospital charts and administrative databases. The primary outcome is a composite of all-cause death or heart failure event. Clinical and imaging predictors of outcome were evaluated on univariable and multivariable analyses.

**Results:**

1028 patients underwent pre-treatment MUGA and 1032 underwent echocardiography. The groups were well matched for most clinical characteristics except patients undergoing MUGA were younger, had more stage 3 breast cancer and more HER2 over-expressing and triple negative cases. Routine follow-up cardiac imaging scan was obtained in 39.3% of patients with MUGA and 38.0% with echocardiography. During a median follow-up of 2448 (1489, 3160) days, there were 194 deaths, including 7 cardiovascular deaths, and 28 heart failure events with no difference in events between the MUGA and echocardiography groups. There were no imaging predictors of the primary composite outcome or cardiac events. Patients without follow-up imaging had similar adjusted risk for the composite outcome compared to those with imaging follow-up, hazard ratio 0.8 (95% confidence interval 0.5,1.3), p=0.457.

**Conclusion:**

The selection of pretreatment echocardiography or MUGA did not influence the risk of death or heart failure in patients with early breast cancer. Many patients did not have any follow-up cardiac imaging and did not suffer worse outcomes. Cardiovascular deaths and heart failure event rates were low and the value of long-term cardiac imaging surveillance should be further evaluated.

## Introduction

Cardiotoxicity is a recognized complication arising from anticancer therapy and may lead to cardiac and cancer morbidity and premature death (1). Cancer treatment regimens containing anthracyclines and/or human epidermal growth factor-2 (HER2) targeted therapies have significant cardiotoxic potential, therefore, patients with breast cancer are particularly vulnerable to adverse cardiac outcomes (2). Anthracyclines can cause direct myocardial damage in dose-dependent fashion and may result in cardiac dysfunction, irreversible cardiomyopathy, and heart failure (1). Trastuzumab, a monoclonal antibody directed against HER2 receptors, has been associated with a two-fold risk of worsening cardiac function and a five-to-seven-fold risk of overt heart failure (3, 4). Monitoring of left ventricular ejection fraction (LVEF) is recommended for the first 12 months in all patients receiving HER2-targeted therapy and/or anthracyclines with a cumulative dose of 250 mg/m^2^ of doxorubicin or equivalent (5). Furthermore, long-term (5+ years) imaging surveillance of cardiac function is recommended for cancer survivors at high-risk for heart failure (4). Cancer treatment related cardiac dysfunction may be prevented and treated with beta blockers and/or renin-angiotensin inhibitors (5–7).

Echocardiography (echo) and multiple-gated acquisition (MUGA) radionuclide ventriculography scans are commonly used modalities to assess cardiac function in patients at high-risk for cancer therapy related cardiotoxicity. Current European Society of Cardiology guidelines recommend echo before MUGA as the first-line modality to assess cardiac function due to a superior safety profile and a more comprehensive cardiac assessment (5). However, in many centers, MUGA is more available and accessible than echo. Previous studies found that MUGA is more sensitive for detecting changes in LVEF than echo (8–10). However, the main disadvantage of MUGA is radiation exposure (~5 to 10 mSv per scan) which is significant in patients undergoing long-term surveillance with repeated exams (1). Furthermore, it has been suggested that patients with cancer are more likely to receive beta-blocker therapy and be referred to a cardiologist for reduced cardiac function detected on echo compared to MUGA (11).

Therefore, we sought to determine if the cardiac imaging modality (echo or MUGA) used for cardiotoxicity surveillance in patients with early breast cancer influences short and long-term cardiovascular outcomes. Furthermore, we sought to understand the pattern of use and duration of cardiac imaging monitoring in these patients.

## Methods

### Patient characteristics

Consecutive adult patients with stage 0-3 breast cancer were identified from a prospective cardio-oncology echocardiography registry at a tertiary cardiac hospital (Mazankowski Alberta Heart Institute, Edmonton, Alberta, Canada) from January 1, 2010, to December 31, 2019. Similar patients undergoing pre-treatment MUGA during the same timeframe were also identified from an affiliated tertiary cancer center (Cross Cancer Institute, Edmonton, Alberta, Canada).

Individual patient charts and electronic health records were reviewed to determine baseline characteristics including prior medical history, cancer type and staging, cancer treatments, cardiac imaging surveillance, and clinical outcomes. Patients with Eastern Cooperative Oncology Group performance status 3 or 4 were excluded. Breast cancer staging was determined according to the TNM classification proposed by the American Joint Committee on Cancer (12). The European Society of Cardiology (ESC) 2022 Heart Failure Association–International Cardio-Oncology Society (HFA-ICOS) risk classification was used to assess the baseline cardiovascular toxicity risk (5). However, previous chemotherapy data and baseline serum biomarkers were unavailable for most patients and were therefore not included in the risk calculation.

The treating medical oncology team ordered cardiac imaging prior to initiating cancer therapy and the modality, echo or MUGA, was usually selected according to availability. As MUGA was scheduled and performed at the cancer center, patients requiring rapid access to cardiac imaging (e.g. stage 3 or neoadjuvant) more commonly underwent this exam. Echocardiograms were acquired at the cardio-oncology clinic using an ultrasonographic system (EPIQ 7C, Philips Medical Systems, N.A., Bothell, USA) equipped with a X5-1 transducer. All patients received echocardiographic contrast (Definity, Lantheus Medical Imaging, North Billerica, USA) bolus regardless of non-contrast image quality in order to minimize variability of LVEF measurements. LVEF was measured from the contrast recordings using the biplane Simpson’s method on commercially available software (13). MUGA scans were performed with technetium 99 m-labeled red blood cells with an activity of approximately 11 to 13 MBq/kg. Images were acquired with a dual-head gamma camera (Siemens Healthineers, Erlangen, Germany and Philips Medical Systems, N.A., Bothell, USA). Scintigrams were smoothed off-line using standard algorithms, and background correction was performed. LVEF was calculated from left ventricular time-activity curves according to the current recommendations (14). For this analysis, patients were assigned either to a MUGA, or echo cohort based on the imaging modality used at baseline. Patients with both imaging modalities at baseline were assigned to the imaging group with the date closest to their cancer diagnosis. Follow-up imaging surveillance strategies were recorded and classified as all echo, all MUGA or mixed modality (both MUGA and echo were performed). Only cardiac imaging performed within 15 months from the baseline scan was included to reflect the standard duration of follow-up at our cancer center. Left ventricular ejection fraction was recorded for each cardiac imaging encounter.

### Outcomes

The primary outcome is a composite of all-cause death and/or new or worsening heart failure diagnosis, and secondary outcomes are (i) cardiovasular death or heart failure event, and (ii) new or worsening heart failure. Events were collected from Jan 11, 2010, to December 31, 2020.

Clinical outcomes data were obtained from medical charts and an independent review of health administrative databases (Alberta Strategy for Patient Oriented Research Support Unit). Information obtained included (i) all admissions to acute care facilities; (ii) all ambulatory encounters, including emergency department visits and (iii) vital statistics including the date and cause of death. Diagnoses were classified using the International Classification of Diseases, Canadian Enhancement; ICD-10. Heart failure events included any new heart failure or cardiomyopathy related encounters during the follow-up period.

Cancer therapy–related cardiac dysfunction (CTRCD) was defined as a reduction in LVEF of ≥10% to a value <50% (5, 15). Cardiac biomarkers and left ventricular strain measurements were not available for many patients and were therefore not used for determining CTRCD events.

### Statistical analysis

The Shapiro-Wilk normality test was used to test the normal distribution of continuous variables and continuous variables were expressed as mean ± standard deviation or median (25^th^, 75^th^ percentile), as appropriate. Categorical variables were expressed as frequency and percentage. Chi-square testing or Fisher’s exact test was used to compare categorical variables between two groups undergoing echo or MUGA at baseline. Two sample t-test or Mann-Whitney U test was used to compare continuous variables among two groups of patients, as appropriate. Univariable Cox proportional regression of outcome was performed in all clinical (cancer-related and cardiovascular disease-related) and imaging metrics at baseline and stepwise forward selection of parameters with p-value<0.2 was used to identify the best predictors of outcome. In the multivariable Cox proportional hazard analysis, all non-collinear 1-year parameters of interest with univariable p-value<0.2 were independently tested for their association with adverse outcomes after adjustment for baseline risk. The Kaplan-Meier method was used to plot time to clinical events for significant parameters from multivariable analysis. A p value less than 0.05 was considered significant for all tests. Statistical analyses were performed using STATA version 17.0 software (StataCorp LP, College Station, Texas).

### Ethics review

This study complies with the Declaration of Helsinki and was approved by the University of Alberta Research Ethics Board (HREBA.CC-16-0511). Informed patient consent was not required due to the minimal risk to the patients involved.

## Results

### Patient characteristics

During the study period, we identified 1028 patients with early-stage breast cancer undergoing pre-treatment MUGA and 1032 patients undergoing pre-treatment echo who fulfilled study entry criteria. Significant baseline differences included older age and prior cancer diagnosis in the echo group and more advanced cancer stage, more aggressive cancer receptor types and more anthracycline and trastuzumab use in the MUGA group (**Table 1**). Patients were well balanced for their baseline cardiovascular risk factors and cardiovascular medications except for more angiotensin-converting enzyme inhibitor use in the MUGA group, and more angiotensin receptor blocker use in the echo group. Baseline HFA-ICOS cardiovascular toxicity risk was similar between cohorts, with 54.1% of MUGA patients and 57.3% of echo patients classified as low risk (**Table 1**).

**Table 1:** Baseline Characteristics.

Variable				MUGA cohort (n=1,028)	Echo cohort (n=1,032)	p-value
Age				53 (47, 61)	55 (48, 62)	0.0071
Female				1019 (99.1%)	1025 (99.3%)	0.610
Body mass index (kg/m^2^)				28.2 (24.0, 32.7)	27.8 (24.2, 32.3)	0.59
Medical History						
Diabetes				90 (8.8%)	98 (9.5%)	0.564
Hypertension				257 (25.0%)	266 (25.8%)	0.696
Dyslipidemia				122 (11.9%)	126 (12.2%)	0.818
Coronary artery disease				10 (1.0%)	20 (1.9%)	0.067
Prior heart failure				7 (0.7%)	10 (1.0%)	0.628
Chronic kidney disease				14 (1.4%)	10 (1.0%)	0.41
Chronic obstructive pulmonary disease				35 (3.4%)	32 (3.1%)	0.694
Previous cancer				60 (5.8%)	98 (9.5%)	0.002
Smoking	Never smoker			549 (53.4%)	588 (56.9%)	0.101
	Current smoker			184 (17.9%)	151 (14.6%)	
	Ex-smoker			295 (28.7%)	294 (28.5%)	
Antiplatelet				52 (5.1%)	73 (7.1%)	0.056
Anticoagulant				7 (0.7%)	13 (1.3%)	0.261
Beta blocker				57 (5.5%)	47 (4.6%)	0.302
ACE-inhibitor				117 (11.4%)	85 (8.2%)	0.016
Angiotensin receptor blocker				80 (7.8%)	118 (11.4%)	0.005
Aldosterone antagonist				1 (0.1%)	4 (0.4%)	0.374
Statin				107 (10.4%)	128 (12.4%)	0.157
Calcium channel blocker				51 (5.0%)	63 (6.1%)	0.259
Diuretic				91 (8.9%)	88 (8.5%)	0.788
Breast Cancer Characteristics						
Stage		0		5 (0.5%)	7 (0.7%)	0.001
		I		126 (12.3%)	144 (13.9%)	
		II		576 (56.0%)	635 (61.5%)	
		III		321 (31.2%)	247 (23.9%)	
Receptor Status		Hormone positive, HER2 negative		512 (49.8%)	591 (57.2%)	0.002
		HER2 positive		404 (39.3%)	357 (34.6%)	
		Triple negative		112 (10.9%)	85 (8.2%)	
Cancer Therapy						
Chemotherapy (any)				958 (93.2%)	902 (87.4%)	<0.001
Anthracycline				538 (52.3%)	510 (49.4%)	<0.001
Trastuzumab				346 (33.7%)	315 (30.5%)	
Anthracycline and trastuzumab				35 (3.4%)	11 (1.1%)	
Other				39 (3.8%)	66 (6.4%)	
Anthracycline dose (mg/m^2^)				312 ± 79	301 ± 57	0.0124
Completed trastuzumab 17 cycles*				357 (93.7%)	298 (91.4%)	0.2612
Hormone therapy				703 (68.4%)	778 (75.3%)	<0.001
Radiation (any)				833 (81.0%)	853 (82.6%)	0.364
Left chest irradiation				415 (40.4%)	418 (40.5%)	0.54
Breast cancer surgery				1017 (98.9%)	1024 (99.1%)	0.645
Left segmentectomy/mastectomy				487 (47.4%)	476 (46.1%)	0.227
Right segmentectomy/mastectomy				457 (44.5%)	489 (47.4%)	
Bilateral mastectomy				61 (5.9%)	47 (4.6%)	
HFA-ICOS risk			Low	556 (54.1%)	592 (57.3%)	0.213
			Moderate	397 (38.6%)	361 (35.0%)	
			High or Very high	75 (7.3%)	80 (7.8%)	

*only patients on trastuzumab included.

All results are expressed as median (25^th^, 75^th^ percentile) or frequency (percentage) except anthracycline dose which is expressed as mean ± standard deviation. MUGA, multi-gated acquisition; ACE, angiotensin converting enzyme; HER2, human epidermal growth factor receptor-2; HFA-ICOS, European Society of Cardiology Heart Failure Association - International Cardio-Oncology Society

### Imaging findings

Baseline LVEF was slightly lower in the MUGA group compared to echo, median LVEF 64% vs. 65%, p = 0.0064 (**Table 2**). At least one follow-up cardiac imaging scan was obtained in 39.3% of patients undergoing pre-treatment MUGA and in 38% with pre-treatment echo within 15 months from the baseline scan. No follow-up imaging was found in 90.4% of patients receiving anthracycline-based treatment compared to 5.1% of patients receiving trastuzumab. Patients who had a baseline MUGA were more likely to be scanned with another imaging modality and had more cardiac imaging tests compared to patients with echo (**Table 2**). The incidence of CTRCD was similar in both groups, 12.2% for MUGA and 12.1% for echo (**Table 2**). For patients with high or very high HFA-ICOS risk, CTRCD occurred in 36% compared to 10.8% and 9.8% for moderate and low risk patients respectively, odds ratio 2.2 (95% confidence interval (1.9, 2.7), p < 0.001.

**Table 2:** Cardiac Imaging Findings.

Variable		MUGA cohort (n=1,028)	Echo cohort (n=1,032)	p-value
Baseline LVEF, %		64 (60, 69)	65 (61, 69)	0.0064
Patients with follow-up imaging within 15 months		404 (39.3%)	392 (38%)	0.556
Median number of baseline and follow-up scans*		6 (5, 6)	6 (5, 6)	0.234
Number of follow-up imaging scans*		1737	1535	0.024
Follow-up imaging modality	Same modality	325 (31.6%)	360 (34.9%)	0.123
	Mixed modality	79 (7.7%)	32 (3.1%)	<0.001
	None	624 (60.7%)	640 (62.0%)	0.556
Cancer therapy related cardiac dysfunction*		125 (12.2%)	125 (12.1%)	0.974
Lowest LVEF, %, at follow-up		58 (54, 61)	58 (55, 62)	0.0427

*Only in patients with follow-up imaging in first 15 months.

All results are expressed as median (25^th^, 75^th^ percentile) or frequency (percentage). MUGA, multi-gated acquisition; LVEF, left ventricular ejection fraction.

### Outcomes

During a median follow-up of 2448 (1489, 3160) days, there were 194 deaths and 28 heart failure events with no difference in events between the MUGA and echo cohorts. The cause of death was cancer related in 171 (88%) cases and cardiovascular related in 7 (3.6%). The 7 cardiovascular related deaths included 4 from coronary artery disease, 1 from arrhythmia, 1 from stroke and 1 from complications of diabetes mellitus. For the 12 patients with late heart failure events after 24 months, 3 were low HFA-ICOS risk at baseline, 5 were moderate risk, 1 was high risk and 3 were very high risk. The timing of cardiac events was also not significantly different between the MUGA and echo groups (**Table 3**).

**Table 3:** Clinical Events.

All-Cause Death or Heart Failure		MUGA cohort (n=1,028)	Echo cohort (n=1,032)	p-value
Duration of follow up (days)		2534 (1487, 3288)	2401 (1529, 3004)	0.0013
Total events*		118 (11.5%)	94 (9.1%)	0.077
Number of events	0-12 months	8 (0.8%)	12 (1.2%)	0.373
	13-24 months	22 (2.1%)	19 (1.8%)	0.627
	24+ months	88 (8.6%)	63 (6.1%)	0.033
Cardiovascular Death or Heart Failure		MUGA cohort (n=1,028)	Echo cohort (n=1,032)	p-value
Duration of follow up (days)		2534 (1487, 3288)	2401 (1528, 3004)	0.0012
Total events		16 (1.6%)	16 (1.6%)	0.991
Number of events	0-12 months	4 (0.4%)	6 (0.6%)	0.753
	13-24 months	5 (0.5%)	5 (0.5%)	1.00
	24+ months	7 (0.7%)	5 (0.5%)	0.579
New Heart Failure		MUGA cohort (n=1,028)	Echo cohort (n=1,032)	p-value
Duration of follow up (days)		2695 (1724, 3372)	2510 (1710, 3123)	<0.001
Total events		15 (1.5%)	13 (1.3%)	0.696
Number of events	0-12 months	3 (0.3%)	5 (0.5%)	0.726
	13-24 months	5 (0.5%)	3 (0.3%)	0.506
	24+ months	7 (0.7%)	5 (0.5%)	0.579

*Only first event was considered.

All results are expressed as median (25^th^, 75^th^ percentile) or frequency (percentage). MUGA, multi-gated acquisition.

**Figure 1:**
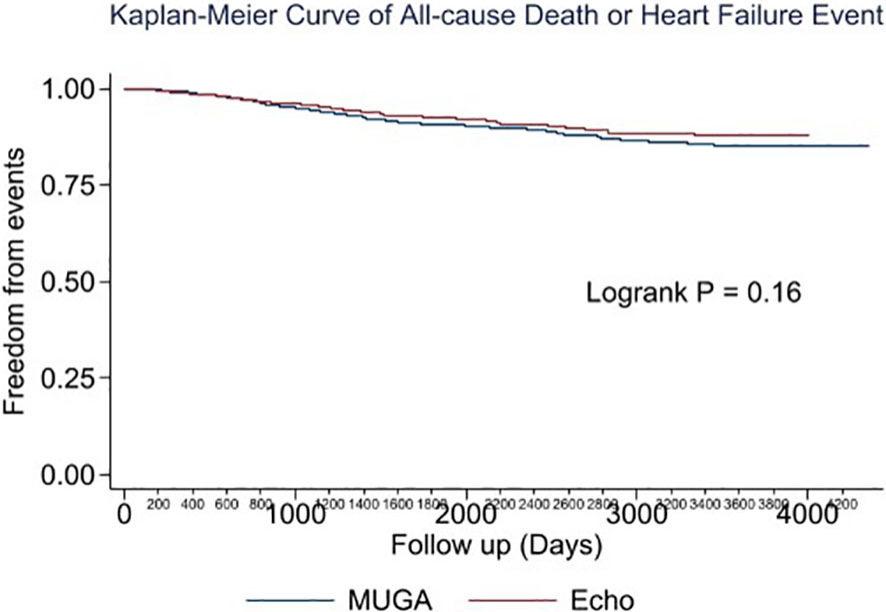
Plot of unadjusted Kaplan-Meier event-free survival for patients with breast cancer assigned to pretreatment multiple-gated acquisition (MUGA) scan (Blue) or echocardiography (Red). The composite event included all-cause death or new heart failure.

**Figure 2:**
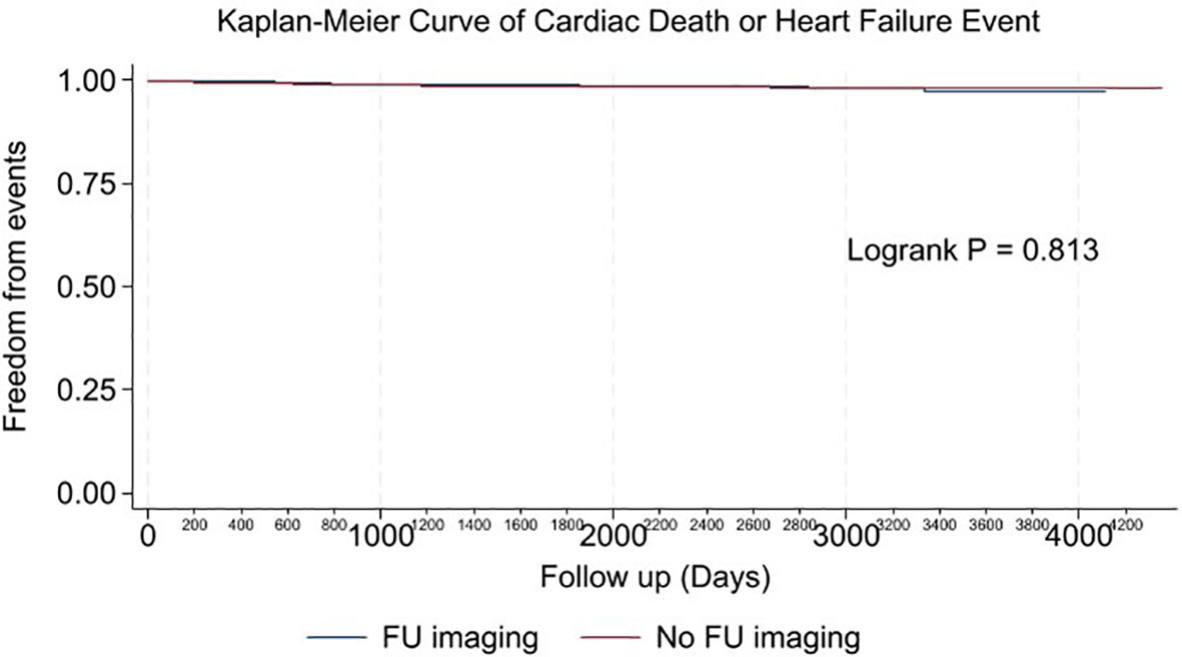
Plot of unadjusted Kaplan-Meier event-free survival curve for cardiac death or new heart failure in patients with imaging follow-up (Blue), and no imaging follow-up (Red).

**Figure 3:**
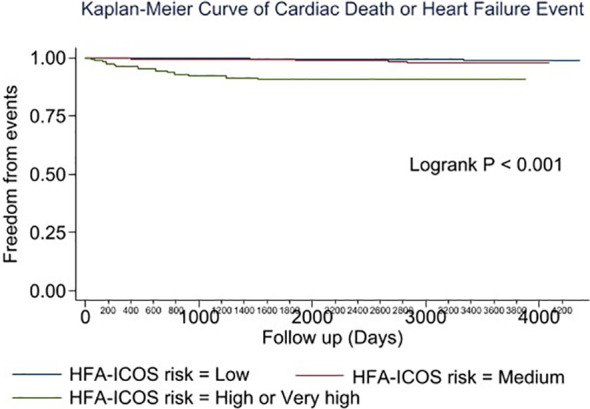
Plot of the adjusted survival curve for cardiac death or new heart failure in patients with breast cancer assigned to low (Blue), moderate (Red), high or very high (Green) European Society of Cardiology Heart Failure Association-International Cardio-Oncology Society risk.

Multivariable analysis identified prior heart failure, chronic kidney disease, chronic obstructive pulmonary disease, aldosterone antagonist therapy, stage 3 breast cancer, triple negative receptor status and absence of cardiac imaging surveillance as predictive of the primary outcome (**Table 4**). The selection of MUGA or echo at baseline was not predictive of clinical events (**Tables 4**–**6**, **Figure 1**). Baseline LVEF and the type of cardiac imaging were not predictive of outcomes (**Tables 4**–**6**). Lack of imaging follow-up was not predictive of adverse outcomes, even after excluding patients receiving non-anthracycline, non-trastuzumab treatments (**Supplemental Tables 1**-**3**) and after excluding low HFA-ICOS risk patients (**Supplemental Tables 4**-**6**). In the overall cohort, risk of cardiac death or heart failure was similar in the 1264 patients without follow-up imaging compared to the 796 patients with follow-up imaging, hazard ratio 0.9 (95% confidence interval 0.5,1.9), p=0.813 (**Figure 2**). However, the HFA-ICOS risk was predictive cardiac death or heart failure on multivariable and adjusted survival analyses (**Table 5** and **Figure 3**).

**Table 4:** Prediction of All-Cause Death or Heart Failure Event- 2060 subjects (212 events).

		Univariable analysis		Multivariable analysis	
		HR (95% CI)	p-value	HR (95% CI)	p-value
Age		1.01 (1.00, 1.02)	0.131		
Body mass index		1.00 (0.98, 1.02)	0.748		
Medical History					
Diabetes		1.4 (0.9, 2.1)	0.143		
Hypertension		1.3 (0.9, 1.7)	0.136		
Dyslipidemia		1.1 (0.7, 1.6)	0.731		
Coronary artery disease		2.1 (0.9, 4.8)	0.071		
Prior heart failure		4.2 (1.9, 9.5)	0.001		
Chronic kidney disease		3.0 (1.3, 6.7)	0.008	2.6 (1.1, 5.9)	0.024
Chronic obstructive pulmonary disease		2.1 (1.2, 3.7)	0.006	1.9 (1.1, 3.3)	0.019
Smoking		1.0 (0.9, 1.2)	0.958		
Beta blocker		1.2 (0.7, 2.1)	0.497		
ACE-inhibitor		1.0 (0.6, 1.6)	0.999		
Angiotensin receptor blocker		1.4 (1.0, 2.2)	0.078		
Aldosterone antagonist		6.6 (2.1, 20.5)	0.001	6.3 (1.9, 20.1)	0.002
Statin		1.0 (0.7, 1.6)	0.853		
Breast Cancer Characteristics					
Cancer stage	0 or 1	Reference			
	2	1.4 (0.8, 2.4)	0.25	1.3 (0.7, 2.3)	0.415
	3	3.9 (2.2, 6.8)	<0.001	3.7 (2.1, 6.6)	<0.001
Receptor status	Hormone positive, HER2 negative	Reference			
	HER2 positive	0.8 (0.6, 1.2)	0.294	1.6 (0.8, 3.1)	0.154
	Triple negative	2.8 (2.0, 3.9)	<0.001	3.0 (2.1, 4.2)	<0.001
Cancer Therapy					
Use of anthracycline therapy		1.17 (0.89, 1.54)	0.251		
Anthracycline dose		1.00 (1.00, 1.00)	0.704		
Number of trastuzumab cycles		0.97 (0.95, 0.99)	0.003	1.0 (0.9, 1.0)	0.064
Left chest irradiation		0.96 (0.71, 1.31)	0.799		
Cardiac Imaging					
Baseline imaging (Echo vs. MUGA)		0.8 (0.6, 1.1)	0.159		
Baseline LVEF, per 1% increase		1.0 (1.0, 1.0)	0.429		
Occurrence of CTRCD		1.2 (0.8, 1.8)	0.264		
Follow-up cardiac imaging	All MUGA	Reference			
	All Echo	0.9 (0.5, 1.4)	0.517		
	Mixed modality	1.4 (0.9, 2.3)	0.136		
	None	0.9 (0.6, 1.3)	0.532		
HFA-ICOS risk	Low	Reference			
	Moderate	1.2 (0.9, 1.6)	0.319	1.0 (0.8, 1.4)	0.961
	High or Very high	2.1 (1.4, 3.3)	<0.001	2.2 (1.4, 3.4)	<0.001

All results are expressed as hazard ratio (95% confidence intervals) or frequency (percentage).

For the composite outcome, the above parameters with univariable P<0.2 underwent stepwise forward selection. The final model includes chronic kidney disease, chronic obstructive pulmonary disease, aldosterone antagonist, cancer stage 3, triple negative receptor status and “high or very high” HFA-ICOS risk. Other parameters are no longer significant in the multivariable model.

HR, hazard ratio; CI, confidence intervals; MUGA, multi-gated acquisition; ACE, angiotensin converting enzyme; HER2, human epidermal growth factor receptor-2; CTRCD, cancer therapy related cardiac dysfunction; HFA-ICOS, European Society of Cardiology Heart Failure Association - International Cardio-Oncology Society.

**Table 5:** Prediction of Cardiovascular Death or Heart Failure Event- 2060 subjects (32 events).

		Univariable analysis		Multivariable analysis	
		HR (95% CI)	p-value	HR (95% CI)	p-value
Age		1.05 (1.02, 1.10)	0.006		
Body mass index		1.06 (1.01, 1.10)	0.009		
Medical History					
Diabetes		4.0 (1.8, 8.6)	<0.001		
Hypertension		2.1 (1.0, 4.2)	0.044		
Dyslipidemia		2.9 (1.3, 6.2)	0.007		
Coronary artery disease		10.4 (3.7, 29.8)	<0.001		
Prior heart failure		19.7 (6.9, 56.2)	<0.001	3.5 (1.1, 11.0)	0.036
Chronic kidney disease		3.1 (0.4, 23.0)	0.261		
Chronic obstructive pulmonary disease		3.1 (0.9, 10.2)	0.061		
Smoking		1.2 (0.8, 1.8)	0.283		
Beta blocker		5.2 (2.2, 12.0)	<0.001		
ACE-inhibitor		2.6 (1.1, 5.9)	0.027		
Angiotensin receptor blocker		1.8 (0.7, 4.7)	0.223		
Aldosterone antagonist		0.0 (0.0, 0.0)	1.000		
Statin		2.6 (1.2, 5.8)	0.018		
Breast Cancer Characteristics					
Cancer stage	0 or 1	Reference			
	2	0.7 (0.2, 1.9)	0.455		
	3	1.2 (0.4, 3.4)	0.749		
Receptor status	Hormone positive, HER2 negative	Reference			
	HER2 positive	0.8 (0.4, 1.8)	0.61		
	Triple negative	1.3 (0.4, 3.8)	0.635		
Cancer Therapy					
Use of anthracycline therapy		0.97 (0.48, 1.94)	0.931		
Anthracycline dose		1.00 (1.00, 1.00)	0.436		
Number of trastuzumab cycles		0.97 (0.93, 1.02)	0.26		
Left chest irradiation		1.12 (0.48, 2.64)	0.795		
Cardiac Imaging					
Baseline imaging (Echo vs. MUGA)		1.0 (0.5, 2.1)	0.92		
Baseline LVEF, per 1% increase		1.1 (1.0, 1.1)	0.025		
Occurrence of CTRCD		3.4 (1.6, 7.2)	0.001		
Follow-up cardiac imaging	All MUGA	Reference			
	All Echo	10.5 (1.4, 79.5)	0.023		
	Mixed modality	17.2 (2.3, 130.6)	0.006		
	None	0.5 (0.0, 5.8)	0.602		
HFA-ICOS risk	Low	Reference			
	Moderate	2.4 (0.9, 6.2)	0.069	2.4 (0.9, 6.2)	0.069
	High or Very high	16.3 (6.6, 40.4)	<0.001	13.0 (4.9, 34.2)	<0.001

All results are expressed as hazard ratio (95% confidence intervals) or frequency (percentage).

For this secondary outcome, parameters with univariable P<0.2 underwent stepwise forward selection. The final model includes prior heart failure and “high or very high” HFA-ICOS risk. Other parameters are no longer significant in the multivariable model.

HR, hazard ratio; CI, confidence intervals; MUGA, multi-gated acquisition; ACE, angiotensin converting enzyme; HER2, human epidermal growth factor receptor-2; CTRCD, cancer therapy related cardiac dysfunction; HFA-ICOS, European Society of Cardiology Heart Failure Association - International Cardio-Oncology Society.

**Table 6:** Prediction of Heart Failure Event- 2060 subjects (28 events).

		Univariable analysis		Multivariable analysis	
		HR (95% CI)	p-value	HR (95% CI)	p-value
Age		1.06 (1.02, 1.10)	0.006		
Body mass index		1.06 (1.01, 1.10)	0.011		
Medical History					
Diabetes		4.8 (2.2, 10.6)	<0.001		
Hypertension		2.3 (1.1, 4.8)	0.033		
Dyslipidemia		3.0 (1.3, 6.7)	0.009		
Coronary artery disease		8.6 (2.6, 28.5)	<0.001		
Prior heart failure		15.4 (4.6, 51.0)	<0.001		
Chronic kidney disease		3.4 (0.5, 25.3)	0.226		
Chronic obstructive pulmonary disease		3.5 (1.1, 11.7)	0.039		
Smoking		1.3 (0.8, 1.9)	0.26		
Beta blocker		5.1 (2.1, 12.6)	<0.001	2.4 (0.9, 6.1)	0.07
ACE-inhibitor		2.5 (1.0, 6.2)	0.045		
Angiotensin receptor blocker		1.6 (0.6, 4.6)	0.378		
Aldosterone antagonist		-- (--, --)	--		
Statin		2.6 (1.1, 6.2)	0.026		
Breast Cancer Characteristics					
Cancer stage	0 or 1	Reference			
	2	0.5 (0.2, 1.4)	0.185		
	3	1.1 (0.4, 3.1)	0.85		
Receptor status	Hormone positive, HER2 negative	Reference			
	HER2 positive	0.7 (0.3, 1.7)	0.499		
	Triple negative	1.4 (0.5, 4.1)	0.566		
Cancer Therapy					
Use of anthracycline therapy		1.0 (0.5, 2.1)	0.948		
Anthracycline dose		1.0 (1.0, 1.0)	0.412		
Number of trastuzumab cycles		0.98 (0.94, 1.03)	0.532		
Left chest irradiation		1.0 (0.4, 2.6)	0.972		
Cardiac Imaging					
Baseline imaging (Echo vs. MUGA)		0.9 (0.4, 1.9)	0.787		
Baseline LVEF, per 1% increase		1.04 (0.98, 1.10)	0.222		
Occurrence of CTRCD		3.6 (1.6, 7.9)	0.002		
Follow-up Cardiac Imaging	All MUGA	Reference			
	All Echo	1.3 (0.4, 4.7)	0.657		
	Mixed modality	1.4 (0.3, 7.7)	0.694		
	None	1.0 (0.3, 3.0)	0.983		
HFA-ICOS risk	Low	Reference			
	Moderate	3.4 (1.2, 9.7)	0.025	3.2 (1.1, 9.2)	0.032
	High or Very high	19.4 (6.8, 55.0)	<0.001	16.0 (5.5, 47.1)	<0.001

All results are expressed as hazard ratio (95% confidence intervals) or frequency (percentage).

For this secondary outcome, the above parameters with univariable P<0.2 underwent stepwise forward selection. The final model includes moderate or “high or very high” HFA-ICOS risk. Other parameters are no longer significant in the multivariable model.

HR, hazard ratio; CI, confidence intervals; MUGA, multi-gated acquisition; ACE, angiotensin converting enzyme; HER2, human epidermal growth factor receptor-2; CTRCD, cancer therapy related cardiac dysfunction; HFA-ICOS, European Society of Cardiology Heart Failure Association - International Cardio-Oncology Society.

## Discussion

In this large, real-world cohort study of patients with early-stage breast cancer, we found that the pretreatment cardiac imaging modality (MUGA or echo) was not associated with all-cause death or new heart failure during extended follow-up. Over 60% of patients had no follow-up cardiac imaging after the baseline scan and lack of imaging surveillance was not associated with adverse cardiac outcomes. HFA-ICOS risk was consistently associated with death, cardiac death and heart failure on multivariable analysis.

### Follow-up imaging and clinical outcomes

ESC cardio-oncology guidelines recommend that asymptomatic patients receiving trastuzumab have follow-up cardiac imaging every 3 months for the first 12 months and repeat testing at 24 months (5). Similarly, patients receiving anthracycline-based chemotherapy should have follow-up cardiac imaging at 12 months and higher risk individuals should undergo 4 intervening scans during and after treatment (5). Additionally, asymptomatic patients at high or very high HFA-ICOS risk are recommended to have follow-up imaging at years 1, 3 and 5 and possibly every 5 years thereafter (5). In this real-world study of patients with early breast cancer receiving cardiotoxic therapy, no follow-up imaging was seen in 61.4%, including 90.4% of patients treated with anthracyclines. Patients with no cardiac imaging follow-up had similar outcomes compared to patients with echo and/or MUGA surveillance, although, heart failure event rates were low. Our findings build on existing knowledge regarding the utility of surveillance cardiac imaging in patients with breast cancer (16, 17). While cardiology involvement in the care of breast cancer patients can lead to adherence to guideline recommended cardiac surveillance during cancer treatment (18), the impact of cardiac monitoring on hard endpoints is unclear. For example, Yu et al. found a lack of association between adherence to routine echocardiogram monitoring and clinical heart failure, suggesting that routine LVEF assessments may be insufficient to decrease the risk of heart failure (17).

In our cohort, we found that patients undergoing echo were more likely to have same modality compared to MUGA (**Table 2**). Although current guidelines recommend using the same imaging method given observed differences in LVEF measurements between different modalities (1, 8, 19), our study did not find that the type of follow-up imaging (same modality or mixed modality) to be a significant predictor of clinical outcomes.

### Comparison with other real-world studies of cardiotoxicity

Among patients with follow-up imaging, 12.2% in the MUGA group and 12.1% in the echo group developed CTRCD. Furthermore, cardiac death or heart failure occurred in only 32 patients (1.6%) during a median follow-up of 6.7 years. The incidence of CTRCD in our cohort is similar to that found in other studies. In the CARDIOTOX registry, which included 865 patients receiving high-risk cancer treatment regimens (84.5% anthracyclines), López-Sendón et al. found the overall incidence of CTRCD was 37.5% (20). However, the majority of the CTRCD were mild (31.6%), defined as asymptomatic patients with LVEF ≥50% with elevated biomarkers or at least one additional abnormal echo parameter, whereas 5.9% of patients had LVEF <50% (20). In another study of 373 patients with breast cancer followed for a mean of 2.4 ± 1 years, there were no cases of LV dysfunction in the anthracycline cohort (0/202), and 16/171 (9%) in the anti-HER2 therapy group (21). In a study by Battisti et al. which included patients with early breast cancer treated with trastuzumab, 5.91% of the patients experienced a LVEF decline ≥ 10% to below 50% but only 5% developed symptomatic heart failure (4.5% with New York Heart Association class II and 0.5% with class III-IV) (22).

### Utility of HFA-ICOS baseline risk stratification

ESC guidelines recommend using HFA-ICOS risk to guide strategies preventing cancer therapy associated cardiotoxicity (5). In our study, patients with high or very high HFA-ICOS risk (7.5% of the overall cohort) were at increased risk for CTRCD as well as cardiac death or heart failure compared to patients with moderate or low risk. This finding is consistent with recent studies that examined the utility of HFA-ICOS proforma in predicting LV dysfunction and heart failure events in patients with breast cancer (21). In a smaller breast cancer cohort, Tini et al. found a similar distribution of HFA-ICOS baseline risk and that patients with increased risk receiving anti-HER2 therapy experienced a greater incidence of LV dysfunction (21). While HFA-ICOS risk predicts cardiac events in patients with breast cancer receiving anthracycline and/or anti-HER2 therapies, future studies should evaluate whether imaging surveillance in higher risk individuals mitigates this risk.

### Limitations

This was a single-center, retrospective study of the relationship between cardiac imaging and clinical outcomes. This design introduces the potential for bias and these results should therefore be confirmed in multicenter, prospective studies. Nevertheless, this is one of the largest studies of cardiotoxicity with a long period of follow-up (median 6.7 years). Heart failure event rates were relatively low compared to other studies of cardiotoxicity in patients with breast cancer. However, it is unlikely that heart failure events were missed given that data was extracted multiple sources including manual chart review and provincial health administrative databases. Referrals to cardiology and the initiation of cardiac medications were not systematically collected in our study and we are therefore not able to determine the potential impact of cardiac imaging on these outcomes. Cardiac biomarkers and left ventricular strain measurements were unavailable for most patients. Therefore, CTRCD was defined using only the LVEF criteria. The role of echo strain to guide management is unclear (23, 24).

## Conclusion

In this contemporary study of patients with early breast cancer undergoing cardiotoxic cancer therapy, the selection of baseline cardiac imaging (MUGA or echo) did not influence the risk of death or heart failure. Many patients did not have any follow-up cardiac imaging and did not suffer worse outcomes. Cardiac death and heart failure event rates were low and the value of long-term cardiac imaging surveillance should be further evaluated.

## Data availability statement

The datasets presented in this article are not readily available because we would require approval from our health research ethics board and possibly a legal agreement to share this data with individuals from outside institutions. Requests to access the datasets should be directed to dpaterson@ottawaheart.ca.

## Ethics statement

The studies involving human participants were reviewed and approved by University of Alberta Health Research Ethics Board. Written informed consent for participation was not required for this study in accordance with the national legislation and the institutional requirements.

## Author contributions

Study conception: KW, SP, JRM, KK, HB, EP, DIP. Data acquisition: KW, SP, MY, WB, AS, HB, DIP. Data analysis: KW, SP, LX, DIP. Manuscript writing and critical review: All. All authors contributed to the article and approved the submitted version.
