# Supplementary material for: Does cardiac imaging surveillance strategy influence outcomes in patients with early breast cancer?
Source: Front Oncol. 2023 Jun 27;13:1168651. doi: 10.3389/fonc.2023.1168651 (PMC10335844; doi:10.3389/fonc.2023.1168651)
Supplement: Supplementary file 1 [file Table_1.docx]

| **Supplemental Table 1. Prediction of All-Cause Death or Heart Failure Event after excluding non-anthracycline and non-trastuzumab cancer treatment - 1955 subjects (199 events)** | | | | | |
| --- | --- | --- | --- | --- | --- |
|  | | Univariable analysis | | Multivariable analysis | |
|  |  | HR(95% CI) | p-value | HR(95% CI) | p-value |
| Age | | 1.01(0.99, 1.02) | 0.321 |  |  |
| Body mass index | | 1.00(0.98, 1.02) | 0.854 |  |  |
| Medical History | | | | | |
| Diabetes | | 1.3(0.9, 2.1) | 0.194 |  |  |
| Hypertension | | 1.2(0.9, 1.6) | 0.236 |  |  |
| Dyslipidemia | | 0.9(0.5, 1.4) | 0.535 |  |  |
| Coronary artery disease | | 2.4(1.0, 5.3) | 0.039 |  |  |
| Prior heart failure | | 2.9(0.9, 9.1) | 0.067 |  |  |
| Chronic kidney disease | | 2.8(1.2, 6.9) | 0.021 | 2.3(0.9, 5.5) | 0.076 |
| Chronic obstructive pulmonary disease | | 1.7(0.9, 3.2) | 0.075 |  |  |
| Smoking | | 1.0(0.9, 1.2) | 0.868 |  |  |
| Beta blocker | | 1.1(0.6, 2.0) | 0.799 |  |  |
| ACE-inhibitor | | 1.0(0.6, 1.6) | 0.987 |  |  |
| Angiotensin receptor blocker | | 1.3(0.9, 2.1) | 0.189 | 9.8(3.1, 31.6) | <0.001 |
| Aldosterone antagonist | | 12.1(3.9, 38.0) | <0.001 |  |  |
| Statin | | 1.0(0.6, 1.5) | 0.842 |  |  |
| Breast Cancer Characteristics | | | | | |
| Cancer stage | 0 or 1 | Reference |  |  |  |
|  | 2 | 1.3(0.7, 2.3) | 0.372 | 1.2(0.7, 2.2) | 0.537 |
|  | 3 | 3.6(2.1, 6.4) | <0.001 | 3.5(2.0, 6.3) | <0.001 |
| Receptor status | Hormone positive, HER2 negative | Reference |  |  |  |
|  | HER2 positive | 0.8(0.6, 1.2) | 0.276 | 1.4(0.7, 2.9) | 0.342 |
|  | Triple negative | 3.0(2.1, 4.2) | <0.001 | 3.2(2.3, 4.6) | <0.001 |
| Cancer Therapy | | | | | |
| Use of anthracycline therapy | | 1.21(0.91, 1.61) | 0.186 |  |  |
| Anthracycline dose | | 1.00(1.00, 1.00) | 0.609 |  |  |
| Number of trastuzumab cycles | | 0.97(0.95, 0.99) | 0.003 | 1.0(0.9, 1.0) | 0.186 |
| Left chest irradiation | | 0.94(0.69, 1.29) | 0.714 |  |  |
| Cardiac Imaging | | | | | |
| Baseline imaging (Echo vs. MUGA) | | 0.8(0.6, 1.1) | 0.126 |  |  |
| Baseline LVEF, per 1% increase | | 1.0(1.0, 1.0) | 0.661 |  |  |
| Occurrence of CTRCD | | 1.1(0.7, 1.7) | 0.623 |  |  |
| Follow-up cardiac imaging | All MUGA | Reference |  |  |  |
|  | All Echo | 0.8(0.5, 1.4) | 0.511 |  |  |
|  | Mixed modality | 0.8(0.4, 1.7) | 0.563 |  |  |
|  | None | 1.3(0.9, 2.0) | 0.183 |  |  |
| HFA-ICOS risk | Low | Reference |  |  |  |
|  | Moderate | 1.1(0.8, 1.5) | 0.589 | 1.0(0.7, 1.3) | 0.777 |
|  | High or Very high | 2.1(1.4, 3.3) | 0.001 | 2.3(1.4, 3.6) | <0.001 |

All results are expressed as hazard ratio (95% confidence intervals) or frequency (percentage).

For the composite outcome, the above parameters with univariable P<0.2 underwent stepwise forward selection. The final model includes angiotensin receptor blocker, cancer stage 3, triple negative receptor status and “high or very high” HFA-ICOS risk. Other parameters are no longer significant in the multivariable model.

Abbreviations: HR = hazard ratio, CI = confidence intervals, MUGA = multi-gated acquisition, ACE = angiotensin converting enzyme, HER2 = human epidermal growth factor receptor-2, CTRCD = cancer therapy related cardiac dysfunction, HFA-ICOS = European Society of Cardiology Heart Failure Association - International Cardio-Oncology Society.

| **Supplemental Table 2. Prediction of Cardiovascular Death or Heart Failure Event after excluding non-anthracycline and non-trastuzumab cancer treatment - 1955 subjects (30 events)** | | | | | |
| --- | --- | --- | --- | --- | --- |
|  | | Univariable analysis | | Multivariable analysis | |
|  |  | HR(95% CI) | p-value | HR(95% CI) | p-value |
| Age | | 1.06(1.01, 1.10) | 0.007 |  |  |
| Body mass index | | 1.06(1.02, 1.11) | 0.003 |  |  |
| Medical History | | | | | |
| Diabetes | | 3.9(1.7, 8.7) | 0.001 |  |  |
| Hypertension | | 2.0(1.0, 4.1) | 0.064 |  |  |
| Dyslipidemia | | 2.8(1.3, 6.3) | 0.012 |  |  |
| Coronary artery disease | | 11.8(4.1, 33.7) | 0.000 | 2.0(0.6, 6.6) | 0.263 |
| Prior heart failure | | 13.6(3.2, 57.1) | 0.000 |  |  |
| Chronic kidney disease | | 3.6(0.5, 26.6) | 0.207 |  |  |
| Chronic obstructive pulmonary disease | | 2.1(0.5, 8.9) | 0.308 |  |  |
| Smoking | | 1.2(0.8, 1.8) | 0.340 |  |  |
| Beta blocker | | 4.6(1.9, 11.3) | 0.001 |  |  |
| ACE-inhibitor | | 2.3(0.9, 5.5) | 0.074 |  |  |
| Angiotensin receptor blocker | | 1.6(0.5, 4.5) | 0.402 |  |  |
| Aldosterone antagonist | | --- | --- |  |  |
| Statin | | 2.5(1.1, 5.8) | 0.035 |  |  |
| Breast Cancer Characteristics | | | | | |
| Cancer stage | 0 or 1 | Reference |  |  |  |
|  | 2 | 0.6(0.2, 1.7) | 0.33 |  |  |
|  | 3 | 1.2(0.4, 3.3) | 0.784 |  |  |
| Receptor status | Hormone positive, HER2 negative | Reference |  |  |  |
|  | HER2 positive | 0.7(0.3, 1.6) | 0.436 |  |  |
|  | Triple negative | 1.4(0.5, 4.1) | 0.574 |  |  |
| Cancer Therapy | | | | | |
| Use of anthracycline therapy | | 0.99(0.48, 2.03) | 0.969 |  |  |
| Anthracycline dose | | 1.00(1.00, 1.00) | 0.449 |  |  |
| Number of trastuzumab cycles | | 0.97(0.93, 1.02) | 0.268 |  |  |
| Left chest irradiation | | 1.12(0.47, 2.63) | 0.801 |  |  |
| Cardiac Imaging | | | | | |
| Baseline imaging (Echo vs. MUGA) | | 1.1(0.5, 2.2) | 0.868 |  |  |
| Baseline LVEF, per 1% increase | | 1.0(1.0, 1.1) | 0.378 |  |  |
| Occurrence of CTRCD | | 2.7(1.2, 6.0) | 0.017 |  |  |
| Follow-up Cardiac Imaging | All MUGA | Reference |  |  |  |
|  | All Echo | 1.8(0.5, 7.3) | 0.391 |  |  |
|  | Mixed modality | 1.9(0.3, 11.3) | 0.489 |  |  |
|  | None | 1.7(0.5, 5.8) | 0.382 |  |  |
| HFA-ICOS risk | Low | Reference |  |  |  |
|  | Moderate | 2.4(0.9, 6.2) | 0.069 | 2.4(0.9, 6.2) | 0.069 |
|  | High or Very high | 15.2(6.0, 38.5) | <0.001 | 12.6(4.6, 34.9) | <0.001 |

All results are expressed as hazard ratio (95% confidence intervals) or frequency (percentage).

For this secondary outcome, parameters with univariable P<0.2 underwent stepwise forward selection. The final model only includes “high or very high” HFA-ICOS risk. Other parameters are no longer significant in the multivariable model.

Abbreviations: HR = hazard ratio, CI = confidence intervals, MUGA = multi-gated acquisition, ACE = angiotensin converting enzyme, HER2 = human epidermal growth factor receptor-2, CTRCD = cancer therapy related cardiac dysfunction, HFA-ICOS = European Society of Cardiology Heart Failure Association - International Cardio-Oncology Society.

| **Supplemental Table 3. Prediction of Heart Failure Event after excluding non-anthracycline and non-trastuzumab cancer treatment - 1955 subjects (27 events)** | | | | | |
| --- | --- | --- | --- | --- | --- |
|  | | Univariable analysis | | Multivariable analysis | |
|  |  | HR(95% CI) | p-value | HR(95% CI) | p-value |
| Age | | 1.02(0.98, 1.07) | 0.007 |  |  |
| Body mass index | | 1.01(0.96, 1.07) | 0.005 |  |  |
| Medical History | | | | | |
| Diabetes | | 2.6(1.1, 6.1) | 0.000 |  |  |
| Hypertension | | 1.1(0.5, 2.5) | 0.067 |  |  |
| Dyslipidemia | | 2.0(0.9, 4.8) | 0.021 |  |  |
| Coronary artery disease | | 4.5(1.4, 15.3) | 0.000 |  |  |
| Prior heart failure | | 8.1(2.4, 27.1) | 0.000 |  |  |
| Chronic kidney disease | | 2.1(0.3, 15.4) | 0.188 |  |  |
| Chronic obstructive pulmonary disease | | 3.1(0.9, 10.4) | 0.251 |  |  |
| Smoking | | 0.9(0.6, 1.4) | 0.297 |  |  |
| Beta blocker | | 3.0(1.1, 8.1) | 0.000 | 0.046 | 0.046 |
| ACE-inhibitor | | 1.5(0.6, 3.7) | 0.038 |  |  |
| Angiotensin receptor blocker | | 0.9(0.3, 2.7) | 0.706 |  |  |
| Aldosterone antagonist | | --- | --- |  |  |
| Statin | | 1.7(0.7, 4.1) | 0.064 |  |  |
| Breast Cancer Characteristics | | | | | |
| Cancer stage | 0 or 1 | Reference |  |  |  |
|  | 2 | 0.3(0.1, 0.9) | 0.149 |  |  |
|  | 3 | 0.8(0.3, 2.3) | 0.884 |  |  |
| Receptor status | Hormone positive, HER2 negative | Reference |  |  |  |
|  | HER2 positive | 0.8(0.3, 2.0) | 0.494 |  |  |
|  | Triple negative | 0.4(0.0, 2.9) | 0.509 |  |  |
| Cancer Therapy | | | | | |
| Use of anthracycline therapy | | 0.70(0.30, 1.59) | 0.851 |  |  |
| Anthracycline dose | | 1.00(1.00, 1.00) | 0.346 |  |  |
| Number of trastuzumab cycles | | 1.00(0.95, 1.05) | 0.488 |  |  |
| Left chest irradiation | | 1.31(0.45, 3.77) | 0.976 |  |  |
| Cardiac Imaging | | | | | |
| Baseline imaging (Echo vs. MUGA) | | 1.0(0.5, 2.3) | 0.985 |  |  |
| Baseline LVEF, per 1% increase | | 1.0(1.0, 1.1) | 0.308 |  |  |
| Occurrence of CTRCD | | 3.1(1.4, 7.1) | 0.007 |  |  |
| Follow-up Cardiac Imaging | All MUGA | Reference |  |  |  |
|  | All Echo | 1.3(0.3, 4.8) | 0.39 |  |  |
|  | Mixed modality | 1.3(0.2, 7.2) | 0.483 |  |  |
|  | None | 0.8(0.3, 2.6) | 0.582 |  |  |
| HFA-ICOS risk | Low | Reference |  |  |  |
|  | Moderate | 3.4(1.2, 9.7) | 0.025 | 3.2(1.1, 9.1) | 0.034 |
|  | High or Very high | 19.3(6.7, 55.6) | <0.001 | 15.7(5.2, 46.8) | <0.001 |

All results are expressed as hazard ratio (95% confidence intervals) or frequency (percentage).

For this secondary outcome, the above parameters with univariable P<0.2 underwent stepwise forward selection. The final model includes beta blocker therapy and moderate or “high or very high” HFA-ICOS risk. Other parameters are no longer significant in the multivariable model.

Abbreviations: HR = hazard ratio, CI = confidence intervals, MUGA = multi-gated acquisition, ACE = angiotensin converting enzyme, HER2 = human epidermal growth factor receptor-2, CTRCD = cancer therapy related cardiac dysfunction, HFA-ICOS = European Society of Cardiology Heart Failure Association - International Cardio-Oncology Society.

| **Supplemental Table 4. Prediction of All-Cause Death or Heart Failure Event after excluding low HFA-ICOS risk patients - 912 subjects (107 events)** | | | | | |
| --- | --- | --- | --- | --- | --- |
|  | | Univariable analysis | | Multivariable analysis | |
|  |  | HR(95% CI) | p-value | HR(95% CI) | p-value |
| Age | | 1.01(0.99, 1.03) | 0.415 |  |  |
| Body mass index | | 1.00(0.97, 1.03) | 0.865 |  |  |
| Medical History | | | | | |
| Diabetes | | 1.3(0.8, 2.0) | 0.303 |  |  |
| Hypertension | | 1.1(0.7, 1.6) | 0.713 |  |  |
| Dyslipidemia | | 0.9(0.6, 1.5) | 0.780 |  |  |
| Coronary artery disease | | 1.8(0.8, 4.2) | 0.152 |  |  |
| Prior heart failure | | 3.7(1.6, 8.4) | 0.002 |  |  |
| Chronic kidney disease | | 2.6(1.0, 6.3) | 0.040 | 3.0(1.2, 7.4) | 0.019 |
| Chronic obstructive pulmonary disease | | 2.4(1.3, 4.5) | 0.005 | 2.7(1.4, 5.1) | 0.002 |
| Smoking | | 0.9(0.8, 1.2) | 0.560 |  |  |
| Beta blocker | | 1.3(0.7, 2.4) | 0.451 |  |  |
| ACE-inhibitor | | 0.9(0.5, 1.4) | 0.606 |  |  |
| Angiotensin receptor blocker | | 1.4(0.9, 2.2) | 0.111 |  |  |
| Aldosterone antagonist | | 5.9(1.9, 18.6) | 0.003 | 7.0(2.1, 23.1) | 0.001 |
| Statin | | 1.0(0.6, 1.6) | 0.927 |  |  |
| Breast Cancer Characteristics | | | | | |
| Cancer stage | 0 or 1 | Reference |  |  |  |
|  | 2 | 0.9(0.4, 1.8) | 0.769 | 0.9(0.4, 1.9) | 0.782 |
|  | 3 | 2.4(1.2, 4.9) | 0.013 | 2.6(1.2, 5.4) | 0.012 |
| Receptor status | Hormone positive, HER2 negative | Reference |  |  |  |
|  | HER2 positive | 0.9(0.6, 1.4) | 0.62 | 2.3(1.1, 5.1) | 0.037 |
|  | Triple negative | 2.5(1.6, 4.2) | <0.001 | 2.4(1.5, 4.0) | 0.001 |
| Cancer Therapy | | | | | |
| Use of anthracycline therapy | | 1.05(0.72, 1.54) | 0.788 |  |  |
| Anthracycline dose | | 1.00(1.00, 1.00) | 0.927 |  |  |
| Number of trastuzumab cycles | | 0.97(0.94, 0.99) | 0.015 | 0.9(0.9, 1.0) | 0.002 |
| Left chest irradiation | | 1.08(0.70, 1.68) | 0.727 |  |  |
| Cardiac Imaging | | | | | |
| Baseline imaging (Echo vs. MUGA) | | 0.8(0.6, 1.2) | 0.367 |  |  |
| Baseline LVEF, per 1% increase | | 1.0(1.0, 1.0) | 0.269 |  |  |
| Occurrence of CTRCD | | 1.5(1.0, 2.5) | 0.071 | 2.1(1.1, 3.7) | 0.016 |
| Follow-up cardiac imaging | All MUGA | Reference |  |  |  |
|  | All Echo | 1.2(0.6, 2.3) | 0.677 |  |  |
|  | Mixed modality | 0.5(0.1, 1.8) | 0.29 |  |  |
|  | None | 1.4(0.8, 2.4) | 0.28 |  |  |
|  | Moderate | Reference |  |  |  |
| HFA-ICOS risk | High or Very high | 1.9(1.2, 2.9) | 0.005 | 1.7(1.1, 2.8) | 0.026 |
|  |  |  |  |  |  |

All results are expressed as hazard ratio (95% confidence intervals) or frequency (percentage).

For the composite outcome, the above parameters with univariable P<0.2 underwent stepwise forward selection. The final model includes chronic kidney disease, chronic obstructive pulmonary disease, aldosterone antagonist, cancer stage 3, HER2 positive receptor status, triple negative receptor status, number of trastuzumab cycles, occurrence of CTRCD and “high or very high” HFA-ICOS risk. Other parameters are no longer significant in the multivariable model.

Abbreviations: HR = hazard ratio, CI = confidence intervals, MUGA = multi-gated acquisition, ACE = angiotensin converting enzyme, HER2 = human epidermal growth factor receptor-2, CTRCD = cancer therapy related cardiac dysfunction, HFA-ICOS = European Society of Cardiology Heart Failure Association - International Cardio-Oncology Society.

| **Supplemental Table 5. Prediction of Cardiovascular Death or Heart Failure Event after excluding low HFA-ICOS risk patients - 912 subjects (25 events)** | | | | | |
| --- | --- | --- | --- | --- | --- |
|  | | Univariable analysis | | Multivariable analysis | |
|  |  | HR(95% CI) | p-value | HR(95% CI) | p-value |
| Age | | 1.03(0.98, 1.07) | 0.269 |  |  |
| Body mass index | | 1.02(0.96, 1.08) | 0.501 |  |  |
| Medical History | | | | | |
| Diabetes | | 2.3(1.0, 5.2) | 0.046 |  |  |
| Hypertension | | 1.1(0.5, 2.4) | 0.818 |  |  |
| Dyslipidemia | | 2.1(0.9, 4.8) | 0.069 |  |  |
| Coronary artery disease | | 5.9(2.0, 17.1) | 0.001 |  |  |
| Prior heart failure | | 11.1(3.8, 32.3) | <0.001 | 3.4(1.1, 10.8) | 0.039 |
| Chronic kidney disease | | 2.0(0.3, 14.7) | 0.502 |  |  |
| Chronic obstructive pulmonary disease | | 2.9(0.9, 9.7) | 0.085 |  |  |
| Smoking | | 0.9(0.5, 1.3) | 0.503 |  |  |
| Beta blocker | | 3.4(1.4, 8.5) | 0.009 |  |  |
| ACE-inhibitor | | 1.6(0.7, 3.8) | 0.293 |  |  |
| Angiotensin receptor blocker | | 1.1(0.4, 2.9) | 0.844 |  |  |
| Aldosterone antagonist | | --(--, --) | --- |  |  |
| Statin | | 1.8(0.8, 4.2) | 0.172 |  |  |
| Breast Cancer Characteristics | | | | | |
| Cancer stage | 0 or 1 | Reference |  |  |  |
|  | 2 | 0.4(0.1, 1.1) | 0.085 |  |  |
|  | 3 | 0.8(0.3, 2.4) | 0.709 |  |  |
| Receptor status | Hormone positive, HER2 negative | Reference |  |  |  |
|  | HER2 positive | 1.0(0.5, 2.3) | 0.951 |  |  |
|  | Triple negative | 0.4(0.1, 3.1) | 0.379 |  |  |
| Cancer Therapy | | | | | |
| Use of anthracycline therapy | | 0.61(0.27, 1.35) | 0.222 |  |  |
| Anthracycline dose | | 1.00(1.00, 1.00) | 0.232 |  |  |
| Number of trastuzumab cycles | | 0.99(0.95, 1.04) | 0.74 |  |  |
| Left chest irradiation | | 1.13(0.41, 3.10) | 0.819 |  |  |
| Cardiac Imaging | | | | | |
| Baseline imaging (Echo vs. MUGA) | | 1.0(0.5, 2.2) | 0.956 |  |  |
| Baseline LVEF, per 1% increase | | 1.0(1.0, 1.1) | 0.08 |  |  |
| Occurrence of CTRCD | | 3.8(1.7, 8.5) | 0.001 |  |  |
| Follow-up cardiac imaging | All MUGA | Reference |  |  |  |
|  | All Echo | 1.6(0.4, 5.5) | 0.495 |  |  |
|  | Mixed modality | 1.3(0.2, 7.1) | 0.759 |  |  |
|  | None | 0.9(0.3, 2.8) | 0.889 |  |  |
|  | Moderate | Reference |  |  |  |
| HFA-ICOS risk | High or Very high | 6.7(3.0, 14.7) | <0.001 | 5.3(2.3, 12.6) | <0.001 |
|  |  |  |  |  |  |

All results are expressed as hazard ratio (95% confidence intervals) or frequency (percentage).

For this secondary outcome, parameters with univariable P<0.2 underwent stepwise forward selection. The final model includes prior heart failure and “high or very high” HFA-ICOS risk. Other parameters are no longer significant in the multivariable model.

Abbreviations: HR = hazard ratio, CI = confidence intervals, MUGA = multi-gated acquisition, ACE = angiotensin converting enzyme, HER2 = human epidermal growth factor receptor-2, CTRCD = cancer therapy related cardiac dysfunction, HFA-ICOS = European Society of Cardiology Heart Failure Association - International Cardio-Oncology Society.

| **Supplemental Table 6. Prediction of Heart Failure Event after excluding low HFA-ICOS risk patients - 912 subjects (23 events)** | | | | | |
| --- | --- | --- | --- | --- | --- |
|  | | Univariable analysis | | Multivariable analysis | |
|  |  | HR(95% CI) | p-value | HR(95% CI) | p-value |
| Age | | 1.02(0.98, 1.07) | 0.359 |  |  |
| Body mass index | | 1.01(0.96, 1.07) | 0.672 |  |  |
| Medical History | | | | | |
| Diabetes | | 2.6(1.1, 6.1) | 0.024 |  |  |
| Hypertension | | 1.1(0.5, 2.5) | 0.817 |  |  |
| Dyslipidemia | | 2.0(0.9, 4.8) | 0.103 |  |  |
| Coronary artery disease | | 4.5(1.4, 15.3) | 0.014 |  |  |
| Prior heart failure | | 8.1(2.4, 27.1) | 0.001 |  |  |
| Chronic kidney disease | | 2.1(0.3, 15.4) | 0.475 |  |  |
| Chronic obstructive pulmonary disease | | 3.1(0.9, 10.4) | 0.069 |  |  |
| Smoking | | 0.9(0.6, 1.4) | 0.651 |  |  |
| Beta blocker | | 3.0(1.1, 8.1) | 0.030 |  |  |
| ACE-inhibitor | | 1.5(0.6, 3.7) | 0.425 |  |  |
| Angiotensin receptor blocker | | 0.9(0.3, 2.7) | 0.872 |  |  |
| Aldosterone antagonist | | --- | --- |  |  |
| Statin | | 1.7(0.7, 4.1) | 0.248 |  |  |
| Breast Cancer Characteristics | | | | | |
| Cancer stage | 0 or 1 | Reference |  |  |  |
|  | 2 | 0.3(0.1, 0.9) | 0.039 |  |  |
|  | 3 | 0.8(0.3, 2.3) | 0.642 |  |  |
| Receptor status | Hormone positive, HER2 negative | Reference |  |  |  |
|  | HER2 positive | 0.8(0.3, 2.0) | 0.658 |  |  |
|  | Triple negative | 0.4(0.0, 2.9) | 0.347 |  |  |
| Cancer Therapy | | | | | |
| Use of anthracycline therapy | | 0.70(0.30, 1.59) | 0.389 |  |  |
| Anthracycline dose | | 1.00(1.00, 1.00) | 0.385 |  |  |
| Number of trastuzumab cycles | | 1.00(0.95, 1.05) | 0.99 |  |  |
| Left chest irradiation | | 1.31(0.45, 3.77) | 0.62 |  |  |
| Cardiac Imaging | | | | | |
| Baseline imaging (Echo vs. MUGA) | | 1.0(0.5, 2.3) | 0.96 |  |  |
| Baseline LVEF, per 1% increase | | 1.0(1.0, 1.1) | 0.588 |  |  |
| Occurrence of CTRCD | | 3.7(1.6, 8.5) | 0.002 | 2.2(0.9, 5.4) | 0.086 |
| Follow-up Cardiac Imaging | All MUGA | Reference |  |  |  |
|  | All Echo | 1.3(0.3, 4.8) | 0.706 |  |  |
|  | Mixed modality | 1.3(0.2, 7.2) | 0.745 |  |  |
|  | None | 0.8(0.3, 2.6) | 0.753 |  |  |
| HFA-ICOS risk | Moderate | Reference |  |  |  |
|  | High or Very high | 5.7(2.5, 12.8) | <0.001 | 4.4(1.8, 10.7) | 0.001 |

All results are expressed as hazard ratio (95% confidence intervals) or frequency (percentage).

For this secondary outcome, the above parameters with univariable P<0.2 underwent stepwise forward selection. The final model includes only “high or very high” HFA-ICOS risk. Other parameters are no longer significant in the multivariable model.

Abbreviations: HR = hazard ratio, CI = confidence intervals, MUGA = multi-gated acquisition, ACE = angiotensin converting enzyme, HER2 = human epidermal growth factor receptor-2, CTRCD = cancer therapy related cardiac dysfunction, HFA-ICOS = European Society of Cardiology Heart Failure Association - International Cardio-Oncology Society.
